# Peritoneal dialysis-associated infection caused by *Mycobacterium abscessus*: a case report

**DOI:** 10.1186/s12882-018-1148-2

**Published:** 2018-11-29

**Authors:** Ryuichi Yoshimura, Miharu Kawanishi, Shungo Fujii, Aska Yamauchi, Kentaro Takase, Kaori Yoshikane, Masahiro Egawa, Hiroaki Shiina, Takafumi Ito

**Affiliations:** 1 Division of Nephrology, Shimane University Hospital, 89-1, Enya-cho, Izumo, Shimane 693-8501 Japan; 2 Department of Urology, Shimane University Faculty of Medicine, 89-1, Enya-cho, Izumo, Shimane 693-8501 Japan

**Keywords:** Peritoneal dialysis, Infection, *Mycobacterium abscessus*, Peritonitis

## Abstract

**Background:**

Peritoneal dialysis (PD)-associated infection caused by *Mycobacterium* spp. is rare. *Mycobacterium abscessus* is one of the most resistant acid-fast bacteria, and treatment is also the most difficult and refractory. Thus, we report a case of PD-associated peritonitis caused by *Mycobacterium abscessus* that was difficult to treat and led to PD failure.

**Case presentation:**

We recently encountered a 56-year-old man who developed PD-associated infection. We initially suspected exit-site infection (ESI) and tunnel infection (TI) caused by methicillin-resistant coagulase-negative *Staphylococcus*. However, antibiotic therapy did not provide any significant improvement. Thus, we performed simultaneous removal and reinsertion of a PD catheter at a new exit site. The patient subsequently developed peritonitis and *Mycobacterium abscessus* was detected in the peritoneal effluent. Thus, the reinserted catheter was removed, hemodialysis was started, and the patient was eventually discharged.

**Conclusions:**

In cases of refractory ESI or TI, it is important to consider non-tuberculous mycobacteria as the potentially causative organism. Even if acid-fast bacterial staining is negative or not performed, detection of Gram-negative bacillus may lead to suspicion and early identification of *Mycobacterium* spp. In PD-associated infection by *Mycobacterium abscessus*, catheter removal is necessary in many cases. Simultaneous removal and reinsertion of the catheter is not recommended, even in cases of ESI or TI. Reinsertion should only be attempted after complete resolution of peritoneal symptoms. After removal of the catheter, careful follow-up is necessary, paying attention to complications such as wound infection, peritonitis, and ileus. In addition, the selection and treatment period of antibiotics in PD-associated infection by *Mycobacterium abscessus* remains unclear, and it is an important topic for future discussion.

## Background

Peritoneal dialysis (PD) is associated with various infectious complications, such as exit-site infection (ESI), tunnel infection (TI), and peritonitis. Various organisms can cause ESI and TI, such as *Staphylococcus aureus* and *Pseudomonas aeruginosa*, which can frequently lead to peritonitis. Thus, these infections must be treated aggressively [1, 2].

Reports of peritonitis caused by non-tuberculous mycobacteria (NTM) are relatively rare, but are becoming more common [3]. More than 50% of the isolates are rapidly growing species, such as *Mycobacterium fortuitum* and *M. chelonae* [4], which are often detected after 3–5 days during routine bacteriological cultures. There is no well-established treatment for NTM-related peritonitis, and personalized treatment should be guided by susceptibility testing [5]. Catheter removal is usually necessary, and experience with non-removal is limited [4, 6, 7]. Unfortunately, most cases develop refractory peritonitis, despite long-term treatment, which ultimately causes PD failure. Thus, we report a case of PD-associated peritonitis caused by *M. abscessus* that was difficult to treat and led to PD failure.

## Case presentation

A 56-year-old Japanese man with end-stage renal disease secondary to diabetic nephropathy visited our hospital because of abdominal pain and pus discharge from the exit site of a PD catheter. He had redness around the exit site and tenderness at the subcutaneous tunnel. The dialysis effluent was not cloudy and the effluent cell count was < 100/μL. Thus, we performed pus swab culture based on a suspicion of ESI and TI. Treatment was started using intravenous vancomycin (1.5 g/day), oral minocycline (200 mg/day), and topical gentamicin ointment, because the patient had a history of ESI caused by methicillin-resistant coagulase-negative *Staphylococcus* (MRCNS).

**Fig. 1:**
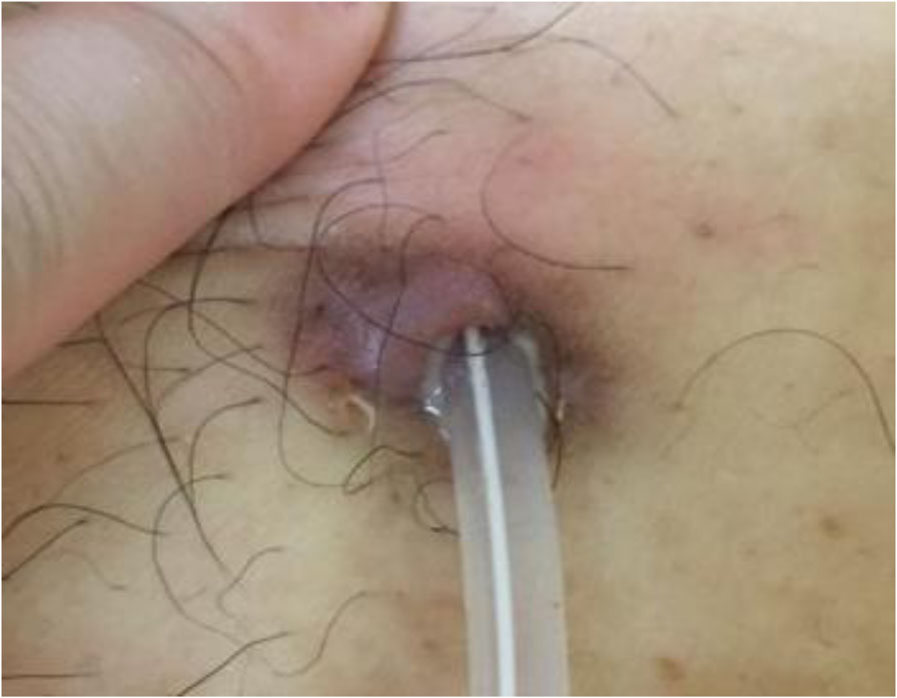
The catheter exit site on the day of admission. Redness and swelling are clearly visible around the catheter exit site

The patient was admitted to our hospital 3 days later, with the following vital signs: blood pressure of 165/104 mmHg, pulse of 86 bpm, and temperature of 36.7 °C. A physical examination revealed continued pus discharge from the exit site, as well as redness and swelling of the surrounding skin (Fig. 1). No rebound tenderness or muscle guarding were observed. A complete blood count from the admission revealed a white blood cell count of 8390/μL, a red blood cell count of 380 × 10^4^/μL, a hemoglobin level of 10.1 g/dL, and a platelet count of 21.4 × 10^4^/μL. The blood test results revealed an albumin level of 2.9 g/dL, a blood urea nitrogen level of 54.3 mg/dL, a creatinine level of 13.95 mg/dL, and a C-reactive protein (CRP) level of 0.09 mg/dL. The white cell count in the dialysis effluent was 7/μL (mononuclear cells: 6/μL, polymorphonuclear cells: 1/μL), and the pus culture revealed the presence of MRCNS.

**Fig. 2:**
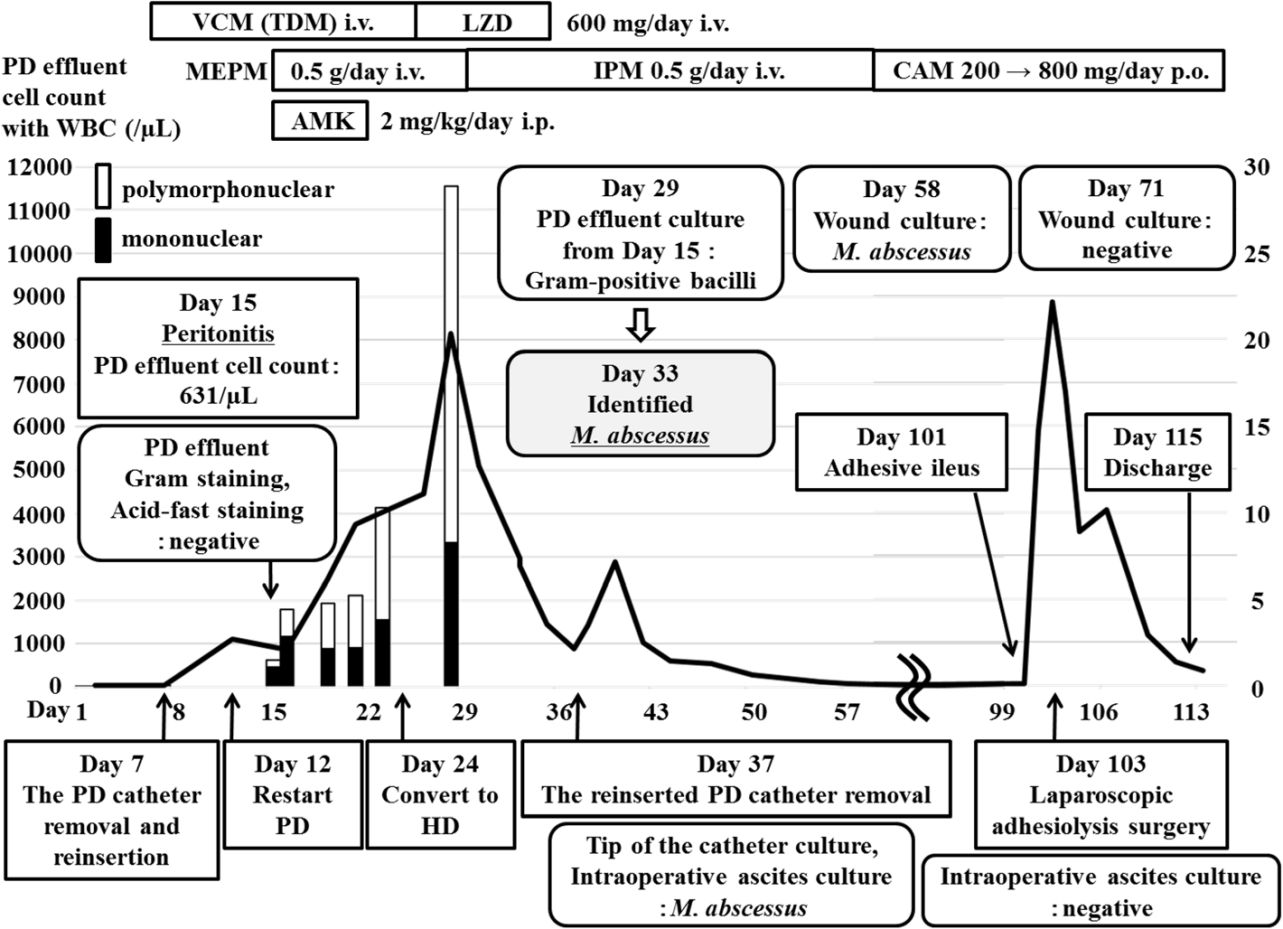
The patient’s course from admission to discharge. AMK: amikacin, CAM: clarithromycin, CRP: C-reactive protein, HD: hemodialysis, IPM: imipenem, LZD: linezolid, *M. abscessus*: *Mycobacterium abscessus*, MEPM: meropenem, PD: peritoneal dialysis, p.o.: oral administration, TDM: therapeutic drug monitoring, VCM: vancomycin

**Fig. 3:**
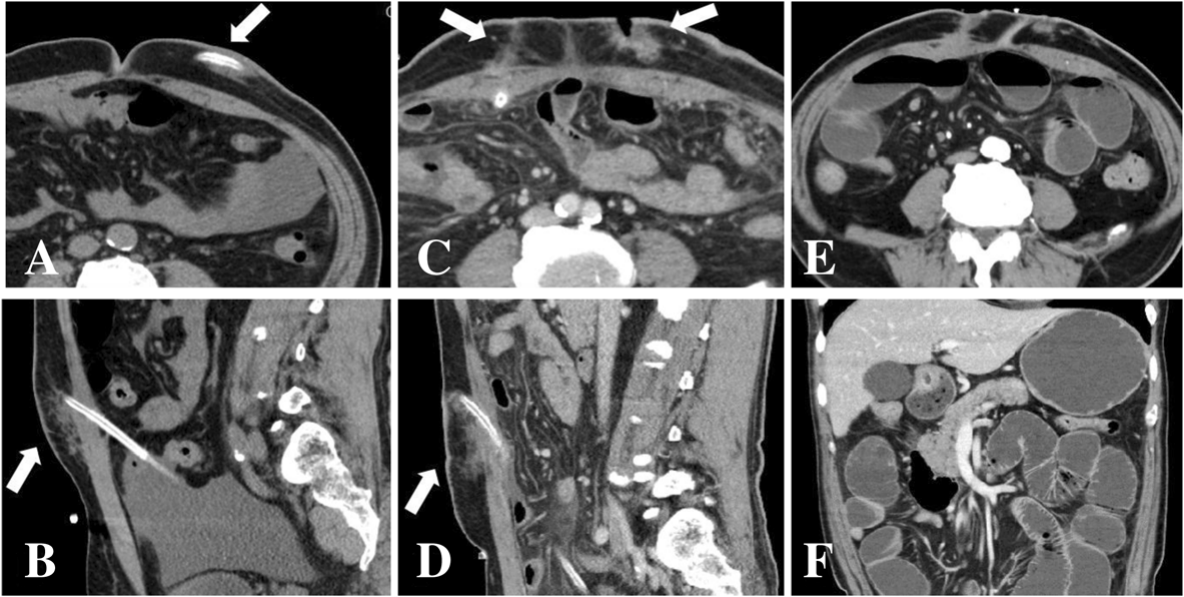
Radiological findings. On day 7, abdominal computed tomography revealed an increased density of fatty tissue around the PD catheter (arrows in **a**, **b**). On day 30, wound dehiscence was observed at the original exit site, with increased density of fatty tissue around the new catheter (arrows in **c**, **d**). On day 101, intestinal dilation was observed (**e**, **f**)

We continued to suspect that the ESI and TI were caused by MRCNS, and continued treatment using intravenous vancomycin (Fig. 2). However, abdominal computed tomography on day 7 revealed an increased density of fatty tissue around the PD catheter (Fig. 3 a, b). Thus, we performed simultaneous removal and reinsertion of the PD catheter at a new exit site, based on the refractory ESI and TI in the absence of peritonitis. The PD was re-started on day 12, although evaluation of the dialysis effluent on day 15 revealed that the white cell count had increased to 631/μL (mononuclear cells: 455/μL, polymorphonuclear cells: 176/μL), which supported a diagnosis of peritonitis. Negative results were obtained from Gram staining and acid-fast staining of the dialysis effluent. The dialysis effluent was then cultured in aerobic, anaerobic, and Ogawa media. Treatment was switched to intravenous meropenem (0.5 g/day) and intraperitoneal amikacin (2 mg/kg/day). On day 23, we observed a rise in the CRP level (10.1 mg/dL) and the number of white cells in the dialysis effluent (4126/μL). Therefore, the patient was converted to hemodialysis (HD) on day 24.

On day 29, gram-positive rods (GPR) were found in the aerobic culture of the dialysis effluent from day 15. These bacilli were sensitive to imipenem but resistant to meropenem and amikacin. On day 30, we noticed growth of acid-fast bacilli in Ogawa medium. The GPR from the aerobic culture were identified as *M. abscessus* on day 33. *M. abscessus* was also detected in cultures of the dialysis effluents from days 20 and 28. After switching the treatment from meropenem to imipenem, the CRP levels decreased. However, ESI and TI persisted at the reinserted catheter and wound dehiscence was detected at the old exit site (Fig. 3 c, d). Thus, the second catheter was removed on day 37, and the patient’s condition subsequently improved. Additional susceptibility testing revealed that the *M. abscessus* was sensitive to clarithromycin, and oral clarithromycin was started after 4 weeks of treatment using imipenem. Cultures of the catheter tip and intraoperative ascites fluid revealed positive results for *M. abscessus*. In addition, pathological findings at the original exit site revealed granulomatous dermatitis and a positive result during acid-fast staining. *M. abscessus* was detected in the wound cultures until day 58, although negative results were observed thereafter. On day 101, the patient developed nausea and vomiting, and computed tomography revealed intestinal dilation (Fig. 3 e, f). On day 103, the patient underwent laparoscopic adhesiolysis surgery because of adhesive ileus, and the intraoperative findings revealed adhesion of the intestinal tract near the abdominal wall site where the catheter had been placed. There were no findings of encapsulating peritoneal sclerosis, and *M. abscessus* was not detected during culture of the intraoperative ascites specimen. The patient was discharged on day 115, completed 6 months of treatment using clarithromycin monotherapy, and did not develop infection or ileus recurrence during the next 2 years.

## Discussion and conclusions

*M. abscessus* belongs to the Ruyon classification group IV of rapidly-growing mycobacteria (RGNTM), which often provide positive results on routine bacteriologic cultures within 7 days. Although *M. abscessus* was previously classified as an *M. chelonae* subspecies, it was reclassified as an individual species in 1992 [8]. This organism is ubiquitous in soil and water, and commonly causes skin, soft tissue, bone, and respiratory infections.

Renaud et al. reported that RGNTM infections accounted for 3% of all culture-positive ESI and PD peritonitis cases [6]. Thus, RGNTM are a rare cause of PD-associated infections. Furthermore, among 57 cases of PD-associated NTM peritonitis, the most prevalent organism was *M. fortinum* (38.6%), which was followed by *M. chelonae* (14.0%), *M. avium* complex (10.5%), and *M. abscessus* (8.8%) [4]. Between 1998 and 2017, there have been 28 reported cases of PD-associated infection caused by *M. abscessus* [6, 7, 9–18], and their characteristics are summarized in Table 1. The reported cases included 16 males and 12 females with a median age of 59 years (range: 14–89). Twenty-one cases (75.0%) involved ESI or TI at the beginning of treatment, which were thought to be para-catheter infections. However, 7 cases (25.0%) involved peritonitis without ESI or TI, which were thought to be transcatheter infections. The large proportion of para-catheter infections may be because mycobacteria are susceptible to heat and ultraviolet light, but can be resistant to disinfectants because they have large amounts of lipids in the cell wall, which can render benzalkonium chloride and chlorhexidine gluconate ineffective. The International Society for Peritoneal Dialysis (ISPD) guidelines recommend daily application of antibiotic cream or ointment to the catheter exit site, because it could prevent ESI caused by *Staphylococcus aureus* and *Pseudomonas* species [5]. However, extensive use of gentamicin ointment for ESIs may predispose patients to NTM infections of the exit site [13, 19]. In the present case, the patient had worked in a forest several times, and had used benzalkonium chloride to clean the exit site, which may have increased the risk of NTM infection.

**Table 1:** Comparison of the present case with previously reported PD-associated infections caused by Mycobacterium abscessus

Reference	Year	Duration of PD	Cause of ESRD, Underlying disease	Infection type at initial treatment	Surgical intervention	Clinical outcome	Antibiotics (duration)
The present case	2017	3 months	DMN	ESI, TI	Simultaneous catheter removal and reinsertion	Removed the reinserted catheter because of developing peritonitis, and converted to HD	CAM (6 months), IPM (4 weeks)
9	2017	12 months	nephrolithiasis	ESI	Simultaneous catheter removal and reinsertion	Removed the reinserted catheter because of prolonged ESI, and converted to HD	CAM, IPM, TGC (N/A)
10	2017	6 months	DMN	ESI	Catheter removal	Paliative care	CAM (44 days), AMK (25 days), IPM (25 days), FRPM (19 days)
11	2015	96 months	N/A	ESI, peritonitis	Catheter removal	Converted to HD, and died after 8 months because of renal hemorrhage and retroperitoneal infection	CAM (165 days), AMK (68 days), MEPM (165 days), LVFX (111 days)
11	2015	70 months	DMN	TI, peritonitis	Catheter removal	Converted to HD	CAM (234 days), AMK (50 days), IPM (70 days), CPFX (217 days), DOXY (180 days)
12	2013	12 months	Herb related	ESI, TI	Debridement Continued PD without catheter removal	Continued PD	CAM (2 months), CPFX (2 months), RFP (2 months)
7	2013	N/A	N/A	peritonitis	Catheter removal	Converted to HD, and died after 5 months because of peritoneal sclerosis	CEZ, GM (N/A)
7	2013	N/A	N/A	peritonitis	Catheter removal	Converted to HD	AMK, CFX, VCM, GM, MFIPC (N/A)
7	2013	N/A	N/A	peritonitis	Catheter removal	Converted to HD	CAM, TIPC/CVA, VCM, GM (N/A)
13	2012	13 months	IgAN, DM, HTN	ESI	Catheter removal	Converted to HD, and reinserted a new PD catheter after 5 months	CAM (28 weeks), AMK (8 weeks), LVFX (4 weeks), MEPM (2 weeks)
13	2012	> 60 months	crescentic mesangioproliferative gromerulonephritis	ESI, peritonitis	Catheter removal	Converted to HD	AMK (8 weeks), CFX (28 weeks), MEPM (4 weeks)
13	2012	49 months	DMN	ESI,	Continued PD without catheter removal	Died after 16 months because of peritonitis	CAM (14 weeks), AMK (14 weeks),
13	2012	19 months	MPA	ESI	Continued PD without catheter removal	Continued PD	CAM (42 weeks), AMK (8 weeks)
13	2012	3 months	obstructive uropathy	peritonitis	Catheter removal	Converted to HD, and reinserted a new PD catheter after 9 months	AMK (4 weeks), AZM (6 weeks), MFLX (20 weeks)
13	2012	> 20 months	DMN	ESI, TI	Catheter removal	Converted to HD	CAM (11 weeks), MEPM (5 weeks)
14	2012	N/A	CGN	ESI, TI	Catheter removal	Converted to HD, and reinserted a new PD catheter after 4 weeks	CAM (14 weeks), IPM (5 weeks), DOXY (9 weeks)
15	2012	60 months	N/A	ESI, peritonitis	Catheter removal	Converted to HD	CAM (8 weeks), AMK (8 weeks)
15	2012	12 months	DMN	peritonitis	Catheter removal	Converted to HD	CAM (3 months)
6	2011	18 months	DM, IHD, HTN	ESI, TI	Catheter removal	Converted to HD	CAM (6 weeks)
6	2011	21 months	DM, HTN	ESI	Untreated	Paliative care	Untreated
6	2011	40 months	HTN	peritonitis	Catheter removal	Converted to HD	CAM (4 weeks), AMK (4 weeks)
6	2011	6 months	DM	ESI, peritonitis	Catheter removal	Converted to HD	CAM (3 months), CPFX (3 months)
6	2011	36 months	DM, IHD, HTN	ESI, TI, peritonitis	Catheter removal	Converted to HD	CAM, AMK (N/A)
6	2011	40 months	DM, IHD, HTN	ESI, TI	Catheter removal	Converted to HD, and reinserted a new PD catheter after 6 weeks	CAM (6 weeks), EB (2 weeks)
6	2011	46 months	DM, IHD, HTN	ESI, TI, peritonitis	Catheter removal	Converted to HD, and reinserted a new PD catheter after 3 months	CAM (3 months), AMK (6 weeks)
16	2007	11 months	CGN	ESI, TI	Simultaneous catheter removal and reinsertion	Removed the reinserted catheter because of developing peritonitis, and converted to HD	CAM (7 weeks), AMK (3 weeks), CPFX (3 weeks)
17	2005	N/A	SLE	ESI	Catheter removal	Converted to HD, and reinserted a new PD catheter after 3 months	CAM (6 weeks), AMK (6 weeks)
18	1998	12 months	DMN	peritonitis	Catheter removal	Converted to HD	CAM (3 months), AMK (3 months)

*AMK* amikacin, *AZM* azithromycin, *CAM* clarithromycin, *CEZ* cefazolin, *CFX* cefoxitin, *CGN* chronic glomerulonephritis, *CPFX* ciprofloxacin, *DM* diabetes mellitus, *DMN* diabetic nephropathy, *DOXY* doxycycline, *EB* ethambutol, *ESI* exit-site infection, *F* female, *FRPM* faropenem, *GM* gentamicin, *HD* hemodialysis, *HTN* hypertension, *IgAN* immunoglobulin A nephropathy, *IHD* ischemic heart disease, *IPM* imipenem, *LVFX* levofloxacin, *M* male, *MEPM* meropenem, *MFIPC* flucloxacillin, *MFLX* moxifloxacin, *MPA* microscopic polyangitis, *N/A* not available, *PD* peritonealdialysis, *RFP* rifampicin, *SLE* systemic lupus erythematosus, *TGC* tigecycline, *TI* tunnel infection, *TIPC/CVA* ticarcillin/clavulanic acid, *VCM* vancomycin,

An official American Thoracic Society/Infectious Diseases Society of America (ATS/IDSA) statement has recommended that surgery is generally indicated for cases of *M. abscessus* infection with extensive disease, abscess formation, or where antibiotic therapy is difficult. Thus, removal of foreign bodies (e.g., percutaneous catheters) is important and probably essential to recovery [20]. Among the 28 reported cases, 24 cases (85.7%) involved catheter removal during treatment, and only 4 cases (14.5%) did not involve catheter removal. Moreover, only 2 patients (7.1%) continued PD during antibiotic treatment or debridement without removing the catheter, and both cases involved ESI or TI without peritonitis. Two other patients died, with 1 patient dying 16 months after developing peritonitis [13], and the other selecting palliative care after developing enterococcal and candidal peritonitis [6]. Among the 24 cases with catheter removal, 23 cases were converted to HD and 1 case was shifted to palliative care. Three cases with ESI or TI but no peritonitis underwent simultaneous removal and reinsertion of the catheter, although all cases were refractory, required subsequent catheter removal, and were ultimately converted to HD. Six other cases received a second catheter after resolution of the peritoneal symptoms and were converted back to PD, with a reinsertion interval of 4 weeks to 9 months. Among the 23 cases with catheter removal and conversion to HD, 1 patient died after 8 months because of renal hemorrhage and retroperitoneal infection, and a second patient died after 5 months because of peritoneal sclerosis [7, 11].

In the studies mentioned above, all patients with peritonitis had their catheters removed. Only two cases of ESI or TI continued PD without progressing to peritonitis, without the removal of the catheter. Hence, there is a possibility to continue PD without catheter removal if *M. abscessus* is identified early and before progressing to peritonitis, provided appropriate antibiotic therapy has started. In this context, acid-fast staining should have been performed to rapidly detect *Mycobacterium* spp*.*, although Song et al. reported that 33.3% of cases involved smear-negative disease [4]. Therefore, in many cases there might be a prolonged delay to identify *M. abscessus*, and the ESI or TI can progress to refractory peritonitis. In our case, MRCNS was detected in the swab culture at the initial visit, but it was thought to be a skin indigenous bacterium from the course. Acid-fast staining of the dialysis effluent was negative at the time of peritonitis diagnosis, but gram-positive bacilli were detected on repeated cultures, and *M. abscessus* was identified. In addition to our case, at least 3 of the 28 reported cases of PD-associated infection caused by *M. abscessus* were initially positive for Gram-positive bacilli, and were diagnosed as *M. abscessus* at a later date. Therefore, we believe that the detection of gram-positive bacillus should alert the clinician to the possible presence of *Mycobacterium* spp., and lead to early identification regardless of acid-fast staining results. In the ISPD guidelines, there is no algorithm to follow if gram-positive bacilli are present, but it is considered to be an important finding in the differentiation of *Mycobacterium* spp.

Many cases may require a prolonged interval to identify *M. abscessus*, and the ESI or TI can progress to refractory peritonitis. Simultaneous reinsertion of a new PD catheter may prolong ESI or TI caused by *M. abscessus*, even in the absence of peritonitis. Thus, catheter reinsertion should only be attempted after the original catheter has been removed and the peritoneal symptoms have completely resolved.

Antibiotic susceptibility testing is recommended in similar cases, as *M. abscessus* has variable susceptibility, although it is uniformly resistant to the standard antituberculous agents [21–23]. According to the ATS/IDSA, serious skin, soft tissue, and bone infections caused by *M. abscessus* should be treated using clarithromycin or azithromycin plus parenteral medication, such as amikacin, cefoxitin, or imipenem [20]. The macrolides are the only oral agents that are reliably active against *M. abscessus* in vitro [22, 24], and the most active parenteral agent is amikacin. However, acquired mutational resistance to clarithromycin and amikacin can occur, because *M. abscessus* only has a single copy of the related gene. The isolate in the present case was sensitive to clarithromycin and imipenem, but was resistant to amikacin. Although the susceptibilities from the reported cases are unclear, 23 cases (82.1%) were treated using clarithromycin and 15 cases (53.6%) were treated using amikacin, with various durations of antibiotic therapy. The ATS/IDSA suggests that a minimum treatment of 4 months is necessary to provide a high likelihood of cure for serious disease, and 6 months of therapy is recommended for bone infections [20, 25]. Thus, although the precise treatment duration remains unclear, several months are likely necessary to successfully treat PD-associated infection. However, due to limited sensitivity to antibiotics and their side effects, 11 cases eventually shifted to monotherapy treatment, of which 9 were oral clarithromycin monotherapy. There was no relapse of infection in 9 cases, and 4 of them resumed PD, supporting the possibility to control infection even with monotherapy treatment. Although clarithromycin is the only effective antibiotic at present, single agent treatment is not desirable, due to the possible induction of resistant bacteria. Even in the ISPD guidelines, there is no statement on the selection of antibiotics and treatment period after sensitivity is determined. Therefore, further studies are necessary to elucidate the optimal treatment regime.

There was a delay in the identification of *M. abscessus* in this case, which we believe was due to the fact that there was a small number of bacteria present at the onset of peritonitis that led to late growth of bacteria in the media, but it is possible that the detection of gram-positive bacillus was effective for the early identification of Mycobacterium spp.. Thus, the simultaneous reinsertion of a new PD catheter might have prolonged the ESI and influenced the progression to peritonitis. Therefore, NTM should be considered as a possible causative organism in cases of refractory ESI or TI. Furthermore, catheter removal is usually necessary in cases of *M. abscessus* PD-associated infection, and reinsertion should only be attempted after complete resolution of peritoneal symptoms after several months of antibiotic therapy. *M. abscessus* is the most resistant bacterium among acid-fast bacteria, and further consideration is needed regarding the selection and treatment period of antibiotics.

## Abbreviations

ATS/IDSA: American Thoracic Society/Infectious Diseases Society of America
CAM: Clarithromycin
CRP: C-reactive protein
ESI: Exit-site infection
GPR: Gram-positive rod
HD: Hemodialysis
ISPD: International Society for Peritoneal Dialysis
MRCNS: Methicillin-resistant coagulase-negative *Staphylococcus*
NTM: Non-tuberculous mycobacteria
PD: Peritoneal dialysis
RGNTM: Ruyon classification group IV of rapidly-growing mycobacteria
TI: Tunnel infection
